# Reduced Efficacy of Fenbendazole and Pyrantel Pamoate Treatments against Intestinal Nematodes of Stud and Performance Horses

**DOI:** 10.3390/vetsci8030042

**Published:** 2021-03-05

**Authors:** Stefania Zanet, Elena Battisti, Federico Labate, Francesca Oberto, Ezio Ferroglio

**Affiliations:** Dipartimento di Scienze Veterinarie, Università degli Studi di Torino, Largo Braccini 2, 10095 Grugliasco, TO, Italy; elena.battisti@unito.it (E.B.); federico.labate@unito.it (F.L.); francesca.oberto@edu.unito.it (F.O.); ezio.ferroglio@unito.it (E.F.)

**Keywords:** strongyles, drug use, drug resistance, equine nematodes

## Abstract

Nematodes are an important cause of disease and loss of performance in horses. Changes in the parasitic fauna of horses have occurred in the past few decades, making cyathostomins the major parasites in adult horses, while large strongyles have become less prevalent. *Parascaris* spp. remains the most important parasite infecting foals and weanlings. Anthelmintic resistance is highly prevalent in cyathostomins and *Parascaris* spp. worldwide and it must be factored into treatment decisions. To assess anthelmintic efficacy in Northern Italy, we sampled 215 horses from 17 sport and horse-breeding farms. Fecal egg count reduction tests (FECRT) were used to assess anthelmintic efficacy. Copromicroscopic analysis was performed using MiniFLOTAC before treatment with fenbendazole, pyrantel pamoate or ivermectin, and repeated 14 days post-treatment. Strongyle-type eggs were detected in 66.91% of horses (CI95% 61.40–73.79%), while *Parascaris* spp. was detected in 2.79% (CI95% 1.94–5.95%). Reduced efficacy against cyathostomins was observed for fenbendazole in 55.56% of the treated animals (CI95% 41.18–69.06%), and for pyrantel pamoate in 75% of animals (CI95% 30.06–95.44%). Ground-based actions must be set in place to promote the uptake of state-of-the-art worm control plans that will prevent clinical disease while minimizing the selection pressure of resistant parasites.

## 1. Introduction

Resistance is a mechanism determined according to genetic base, defined as the ability of a parasite within a population to survive treatments that are generally effective against the same species and stage of infection [1]. Today, anthelmintic resistance in horses is a worldwide phenomenon involving all major equine parasites [2] which has arisen as a clinical and economic issue [3]. Three major categories of broad-spectrum anthelmintics are used worldwide for anthelmintic treatments in horses, namely imidazothiazoles/tetrahydropyrimidines, benzimidazoles and macrocyclic lactones [4]. Regular interval treatment protocols aiming to control migrating strongyles have contributed since the 1960s to greatly reducing the prevalence of *Strongylus* spp. [5], as a result making cyathostomin nematodes the most prevalent parasites in adult horses worldwide [6]. Among others, strategic treatment of all horses without previous assessment of Faecal Egg Count (FEC) has helped to trigger the emergence of anthelmintic resistance [7]. The first indications of resistance were reported for phenothiazine against cyathostomin nematodes in 1960 [8], and nowadays, resistance to all three broad-spectrum anthelmintics has been reported in ruminants, horses and companion animals [2,9,10]. While resistance to benzimidazoles and tetrahydropyrimidines has been increasing since the early 1990s, reaching 100% prevalence in some countries [11,12,13], macrocyclic lactones showed full efficacy until the past few years, when cases of resistance started to be reported [14,15,16,17]. The evidence of a widespread resistance against broad-spectrum anthelmintic drugs represents an important issue. Larval cyathostominosis is caused by the synchronous emergence of encysted small strongyle larvae, which, despite being rarely observed, causes severe intestinal syndromes in both foals and adult horses [18]. In fact, even though foals are generally more susceptible due to their lower immune response, the susceptibility can be lifelong and adult horses could experience clinical symptoms of infection [18]. Animal management influences the risk of infection. Previous studies have observed a higher level of infection in horses from stud farms and facilities with high traffic of animals [19], compared to stallions, which often graze alone, and animals predominantly stabled (i.e., riding schools, boarding stables), where the risk of infection is reduced [20].

The fecal egg count reduction test (FECRT) is recognized as the gold standard for defining the anthelmintic susceptibility of both *Parascaris* spp. and cyathostomin infections in the field [2]. We therefore used FECRT with the aim of assessing the occurrence and diffusion of anthelmintic efficacy in stud and performance horses in a previously uninvestigated area in Northern Italy. 

## 2. Materials and Methods 

A questionnaire was administered to the farm manager/veterinarian prior to enrollment to establish history of anthelmintic drug usage. Specifically, we collected information on frequency of treatment, active principles used as well as treatment criteria (selective vs. strategic vs. symptomatology-based treatment) (Table 1).

Only facilities for which detailed information on anthelmintic drug usage was available for the 3 years prior to the beginning of the study were included. Eligible facilities must have used the same anthelmintic product for 2 consecutive treatments to be enrolled. From each facility, 25% of the horses present (minimum enrolled horses *n* = 2, maximum enrolled horses *n* = 39, proportionally divided by age class) were subjected to FECRT [21,22] to evaluate anthelmintic efficacy. Sampled horses did not receive treatment within 8 weeks prior to FECRT. In the period between April and October, feces were collected from the rectum of each horse, preserved airtight at +4 °C and analyzed at the Department of Veterinary Sciences, University of Turin Italy within 48 h from collection. Full ethical and institutional approval was given by the Department of Veterinary Sciences, University of Turin (Italy). Quantitative copromicroscopic analysis was performed using MiniFLOTAC (sensitivity 5 EPG) [23] with zinc sulfate floatation solution (specific gravity at 75 °C: 1.366–1.394). For each horse, fecal egg count (FEC) was performed just before treatment (T0) and again 14 days later (T14). The egg count reduction (FECR) was calculated according to the following formula:

$$FECR\left(\%\right)=\frac{\left(EPG\;T_{0}-EPG\;T_{14}\right)*100}{EPG\;T_{0}}$$

To establish treatment efficacy, we used the cutoff values (mean percent reduction in FEC) suggested for each drug category by Nielsen et al. [1]. Horses were treated with fenbendazole (FBZ), pyrantel pamoate (PYR) or ivermectin (IVM) with dosages recommended by manufacturers. The anthelmintic drug used for FECRT was the same used in each facility for the previous 2 treatments. To ensure the correct administration of treatment, horses were individually weighed and veterinary practitioners administered the treatment. 

Coprocultures were performed on all positive samples at T0 and repeated, if positive, at T14 post-treatment. Individual samples (10 g) were incubated in Petri dishes for 10 days in a stove at 23–25 °C to obtain infective L3 larvae. The larvae were isolated using the Baermann technique and 10 larvae per sample were identified to larval type by morphology [24,25,26,27]. Total genomic DNA of L3 larvae was extracted using the GeneElute™ Mammalian Genomic DNA Miniprep Kit (Sigma-Aldrich International GmbH, Buchs, Sankt Gallen, Switzerland) and amplified using primers for the beta-tubulin codon 200, as described by von Samson-Himmelsrjerna [28]. Primer combination for the benzimidazole-sensitive allelic variant CN24FS/CN30R was used in parallel with the primer pair CN25FR/CN30R to differentiate the codon 200 TTC/TAC polymorphism in the small-strongyle beta-tubulin gene. Positive (DNA extracted from resistant strongyle larvae) and negative controls were included in each PCR reaction and all necessary measures were taken to minimize the risk of contamination. Samples of L3 from animals treated with FBZ were tested by PCR individually, while samples of animals from farms using anthelmintic products other than FBZ were homogeneously pooled by farm and drug used, prior to PCR.

Statistical analysis was performed using R3.4.4 [29]. Chi-square tests, (X^2^) confidence intervals at 95% and odds ratio (OR) and generalized linear models (GLM) were used to assess the influence of management practices and horse individual characteristics on parasite load and reduced efficacy. Differences were considered significant at *p* < 0.05.

## 3. Results

Seventeen facilities were enrolled in the study program, namely 2 stud farms, 1 racecourse and 14 livery yards. All selected facilities treated all horses on a strategic basis, with varying frequency between 3 and 6 months. The most commonly used drug was IVM (*n* = 166 horses from 13 facilities), followed by FBZ (*n* = 45 horses from 3 facilities) and PYR (*n* = 4 horses from 1 facility). A total of 215 horses were sampled (mean *n* = 13 subjects/farm, sd = 12). The horses were 38% geldings, 13% stallions and 49% mares and were distributed in three age classes (*n* = 7 < 1 years old, *n* = 35 1 ≤ x > 3 years, *n* = 173 ≥ 3 years old). FEC at T_0_ detected intestinal strongyle eggs in 66.91% of the tested animals (CI95% 61.40–73.79%). Infection in each positive facility ranged from 16.67% (CI95% 4.70–44.8%) to 100% (CI95% 34.24–100%) while all horses from one recreational center tested negative (Table 2). 

**Figure 1:**
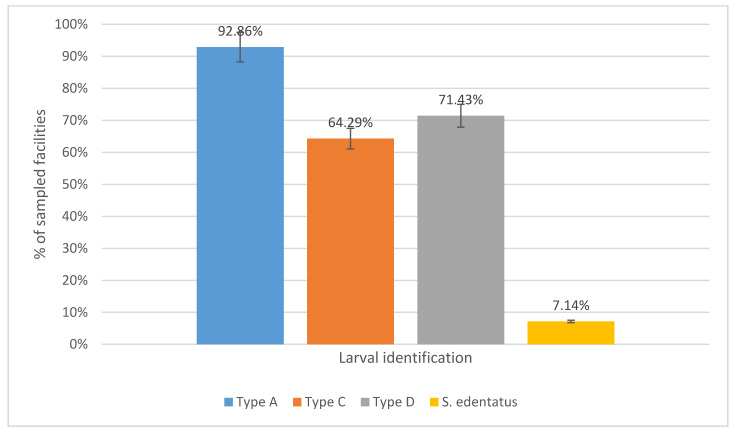
Total number (%) of facilities in which each of the identified larval types or *S. edentatus* were reported.

No significant differences were detected among subjects of different age classes (*p* > 0.05), while management was shown to highly influence parasite load. Horses housed exclusively on paddocks showed greater FEC than those housed in boxes (X^2^ = 12.35, *p* < 0.05, OR = 2.77) or on pastures (X^2^ = 4.89, *p* < 0.05, OR = 1.41). Frequent (once or more per week) manure removal was significantly associated with lower FEC (GLM, *p* < 0.05). The mean EPG value for strongyle eggs at T_0_ was 515 (min = 0, max = 1615, sd 497; Table 2). *Parascaris* spp. eggs were detected in six subjects (*p* = 2.79%; CI95% 1.94–5.95%) from four facilities (1 racecourse, 1 breeding center and 2 recreational facilities). As expected, animals under 1 year of age were significantly more infected with *Parascaris* spp. compared to older age categories (X^2^ = 4.98; *p* < 0.05) and *Parascaris* spp. was detected in 100% of the facilities where foals were present. *Parascaris* spp. FECRT results showed a 100% reduction in all infected animals. In animals treated with PYR, the efficacy of treatment against strongyles yielded an FECR lower than the expected cutoff [1] in 75% of animals (CI95% 30.06–95.44%), while full efficacy (100% strongyle FECR) was observed in animals treated with IVM. Among the 45 animals treated with FBZ, 55.56% (CI95% 41.18–69.06, 25/45) showed an FECR for strongyle eggs that was lower than 90%. Reduced efficacy was detected only in animals from one of the three facilities where FBZ was used, namely in stud farm 2, while FECRT from the other two livery yards using FBZ was above the 99% expected cutoff value for efficacy. High treatment frequency was highly associated with reduced efficacy. Reduced efficacy was detected only in stud farm 2, which strategically treated animals every 3 months (GLM, *p* < 0.05). PCR for the beta-tubulin codon 200, differentiating the codon TTC/TAC polymorphism, carried out on FBZ-resistant horses from stud farm 2, showed the presence of the resistant homozygote variant (RR) in 20 subjects, while the remaining five showed the heterozygote variant (Rr). PCR of pooled samples allowed determination of the presence of the resistance allele R in all samples from all studied facilities. Coproculture of positive T_14_ samples allowed the identification of three different cyathostomin larval types, namely type A (including *Cylicocyclus nassatus* (NAS)*, Cylicostephanus goldi* (GLD), *Cyathostomum catinatum* (CAT), *Cylicostephanus longibursatus* (LNG), *Cylicocyclus insigne* (INS) and *Coronocyclus coronatus* (COR)), type C (including *Cyathostomum pateratum* (PAT) and *Cylicostephanus calicatus* (CAL)), and type D including *Cylicocyclus ashworthi* (ASH), while *Strongylus edentatus* (EDN) was detected in one individual sample. For each of the identified larval types at T_0_, we reported the distribution among sampled facilities (Table 3) and summarized the proportion of infected positive facilities (Figure 1).

## 4. Discussion

The management of equine intestinal nematodes has always been a challenge for veterinarians worldwide. Following the rise in anthelmintic resistance and the lack of new treatment options, gold-standard strategies for parasite control have evolved from being drug-centered to relying on environmental management practices (e.g., pasture hygiene, regular monitoring of FEC), and on the implementation of selective treatment strategies to reduce selection pressure for resistance [2]. Guidelines for parasite control elaborated by veterinary parasitologists and equine practitioners worldwide are useful tools to mitigate the impact of anthelmintic resistance (i.e., European Scientific Counsel Companion Animal Parasites, American Association Equine Practitioners) [30,31]. Despite the resonance of growing resistance, owner uptake of evidence-based anthelmintic treatment protocols is still low and owners are reluctant to implement more sustainable practices for parasite control [16,32,33,34]. All enrolled facilities practiced calendar-based anthelmintic treatments without previous assessment of FEC.

IVM was the most frequently used anthelmintic product, as it was used by 12 recreational facilities and by one breeding center. *Parascaris* spp. showed no resistance, regardless of the type of anthelmintic used. For strongyles, FECRT evidenced no resistance phenomena in any of the facilities where macrocyclic lactones were used. In the literature, recent studies detected reduced efficacy of IVM in horses from the UK [16], Italy [16,35], the Netherlands [35], Belgium [35] and Finland [36]. FBZ was used in two livery yards and in one stud farm. In this last facility, the mean strongyle FECR value was 70.24%. PYR, used only in one of the enrolled facilities, showed values of strongyle FECR lower than the threshold limit for resistance in 75% of the horses. Previous studies detected resistance and/or suspected resistance in cyathostomins from Italian horse yards against PYR and FBZ [16,37]. In Europe, benzimidazole resistance was widely detected in horses from Ukraine [38], the UK [16,39], Germany [16], Switzerland [40], Sweden [41], Denmark [42] and the Slovak Republic [43], while resistance to PYR has been previously reported in Norway [12], Sweden [44], Denmark [45], the UK [16], Finland [36] and Germany [16].

Molecular determination of FBZ resistance-related alleles allowed determination of the presence of resistance allelic variants in all enrolled facilities, including those where FBZ had not been used for at least 3 years. Once acquired, resistance is permanent, even in populations of cyathostomins that remain unexposed to anthelmintic treatment for decades [46]. This finding underlines the importance of molecular-based approaches for early diagnosis of resistance [28], as extensively reported in the literature [47]. FBZ resistance was directly associated with frequency of treatment: horses treated every 3 months were more likely to host resistant strongyles (GLM, *p* < 0.05).

Our study accurately demonstrated the reduced efficacy of FBZ and PYR against strongyles, although some limitations are present. As previously reported from other countries [35], horse facilities investigated in this study tended to host a limited number of animals. Even though this clearly depicts the reality of the majority of the horse farms in Northern Italy, further studies are needed in order to investigate the occurrence of anthelmintic resistance in larger groups of horses.

We observed higher prevalence of strongyle eggs in horses housed in paddocks if compared to those housed in boxes and pasture. Access to grass (either pasture or paddock) has been only weakly associated with cyathostomin infection [18] and individually housed horses are reported to be at lower risk of infection. We underline the importance of housing and pasture hygiene in maintaining a lower burden of infection [48,49,50]. Moreover, recent studies have shown the possibility for equine cyathostomins to develop to infective larvae on straw bedding [51], so more studies are required to further investigate the burden of different management practices.

Given the increasing importance of anthelmintic-resistant cyathostomins in horse management, it will be essential to promote the uptake of sustainable practices among veterinary practitioners, farm managers and horse owners in order to improve the collaboration between them and to establish an integrated parasitic control plan based on the correct use of anthelmintic drugs, adequate pasture hygiene and periodic FEC analysis.

## Figures and Tables

**Table 1:** Anthelmintic drug usage (treatment criteria, frequency and active principle) is reported for each facility (N = number of facilities) enrolled in the study.

Treatment Method	Facilities (N)
Strategic	13
Strategic + symptomatology	4
Selective	0
Frequency	
Every 3 months	2
Every 4 months	4
Every 6 months	11
Active Principle	
Ivermectin	13
Fenbendazole	3
Pyrantel Pamoate	1

**Table 2:** For each of the 17 facilities enrolled in the study, we report the total number of sampled subjects, the number of subjects positive for strongyle eggs at T0, prevalence of infection, strongyle fecal egg count at T0 (mean egg per gram value, EPG), *Parascaris* spp. egg count (mean egg per gram value, EPG) and type of treatment (fenbendazole (FBZ), pyrantel pamoate (PYR) or ivermectin (IVM)).

Facility	Total Sampled	Positive	Prevalence (IC95%)	Strongyles FEC (Mean EPG)	*Parascaris* Egg (Mean EPG)	Anthelmintic Treatment
Stud farm 1	8	8	100.00% (67.56–100)	1538		IVM
Stud farm 2	28	26	92.86% (77.35–98.02)	620		FBZ
Racecourse	11	8	72.73% (43.44–90.25)	745	36	IVM
Livery yard 1	21	11	52.38% (32.37–71.66)	265		IVM
Livery yard 2	2	2	100.00% (34.24–100)	250		IVM
Livery yard 3	37	30	81.08% (65.80–90.52)	525	980	IVM
Livery yard 4	10	2	20.00% (5.67–50.98)	90		IVM
Livery yard 5	4	4	100.00% (51.01–100)	225		PYR
Livery yard 6	10	5	50.00% (23.66–76.34)	140		IVM
Livery yard 7	11	6	54.55% (28.01–78.73)	255	9	FBZ
Livery yard 8	7	3	42.86% (15.82–74.95)	100		IVM
Livery yard 9	39	29	74.36% (58.92–85.43)	340		IVM
Livery yard 10	6	6	100.00% (60.9–100)	1615		FBZ
Livery yard 11	12	2	16.67% (4.70–44.80)	50	50	IVM
Livery yard 12	2	2	100.00% (34.24–100)	750		IVM
Livery yard 13	2	2	100.00% (34.24–100)	1250		IVM
Livery yard 14	5	0	0.00% (0.00–43.45)	0		IVM

**Table 3:** Numbers of individuals within each larval type and strongyles in each of the enrolled facilities at T0. In each facility, for every positive horse, we morphologically identified 10 L3 specimens.

Facility	Type D	Type C	Type A	*S. edentatus*
Stud farm 1	0	7	73	0
Stud farm 2	21	34	205	0
Racecourse	7	2	68	4
Livery yard 1	5	0	105	0
Livery yard 2	1	0	19	0
Livery yard 3	0	60	240	0
Livery yard 4	2	2	16	0
Livery yard 5	3	0	26	0
Livery yard 6	10	5	35	0
Livery yard 7	0	0	60	0
Livery yard 8	2	6	24	0
Livery yard 9	0	0	290	0
Livery yard 10	3	5	54	0
Livery yard 11	0	0	18	0
Livery yard 12	0	0	20	0
Livery yard 13	1	1	18	0
Livery yard 14	0	0	0	0

## Data Availability

All data are available in the text.
